# Genome-wide association study reveals novel loci associated with feeding behavior in Pekin ducks

**DOI:** 10.1186/s12864-021-07668-1

**Published:** 2021-05-08

**Authors:** Guang-Sheng Li, Feng Zhu, Fan Zhang, Fang-Xi Yang, Jin-Ping Hao, Zhuo-Cheng Hou

**Affiliations:** 1 National Engineering Laboratory for Animal Breeding and Key Laboratory of Animal Genetics, Breeding and Reproduction, MARA; College of Animal Science and Technology, China Agricultural University, Beijing, 100193 China; 2 Beijing Golden Star Inc, Beijing, 100076 China

**Keywords:** Pekin duck, Feeding behavior, Genome-wide association analysis

## Abstract

**Background:**

Feeding behavior traits are an essential part of livestock production. However, the genetic base of feeding behavior traits remains unclear in Pekin ducks. This study aimed to determine novel loci related to feeding behavior in Pekin ducks.

**Results:**

In this study, the feeding information of 540 Pekin ducks was recorded, and individual genotype was evaluated using genotyping-by-sequencing methods. Genome-wide association analysis (GWAS) was conducted for feeding behavior traits. Overall, thirty significant (*P*-value < 4.74E-06) SNPs for feeding behavior traits were discovered, and four of them reached the genome-wide significance level (*P*-value < 2.37E-07). One genome-wide significance locus associated with daily meal times was located in a 122.25 Mb region on chromosome 2, which was within the intron of gene ubiquitin-conjugating enzyme E2 E2 (*UBE2E2)*, and could explain 2.64% of the phenotypic variation. This locus was also significantly associated with meal feed intake, and explained 2.72% of this phenotypic variation.

**Conclusions:**

This study is the first GWAS for feeding behavior traits in ducks. Our results provide a list of candidate genes associated with feeding behavior, and also help to better understand the genetic mechanisms of feeding behavior patterns in ducks.

**Supplementary Information:**

The online version contains supplementary material available at 10.1186/s12864-021-07668-1.

## Background

Animal behavior refers to the instant response of animals to changes in their living environment or variations in their internal physiological conditions. Therefore, feeding behavior is of great significance for maintaining individual survival and population development. Feeding behavior patterns have been studied intensively in different animals, including mice [1], zebra finch [2], monkeys [3], pigs [4] and cows [5], which can be helpful for constructing a physiological model for animal health and production. In recent years, meat ducks have become one of the main source of animal proteins in Asian countries. Pekin ducks are an ideal model to examine the effects of genetic selection due to their history of intensive artificial selection [6, 7].

Feeding behavior traits are also very vital in Pekin ducks. For one thing, feeding behavior can be an effective way to track animal performance and health status. For another thing, researchers had found that there were strong associations between feeding behavior and feed efficiency in broilers and ducks [8, 9]. Nowadays, with the widespread use of automatic feeders, individual feeding behavior can be recorded accurately in a large population, which could facilitate the understanding of genetic bases for feeding behavior in poultry. In our previous study, we found that the feeding tendency of Pekin ducks was obviously different at different residual feed intake (RFI) levels [10]. Meanwhile, we also showed that feeding behavior traits were highly heritable in Pekin ducks. Certain feeding behaviors had the potential to improve other economic traits, with an improvement in feed efficiency [11]. However, few QTLs related to feeding behavior traits have been reported in ducks due to the non-availability of genotyping arrays.

The goal of this study was to investigate the genetic markers associated with feeding behavior in Pekin ducks. Previous researchers had shown that genotyping-by-sequencing (GBS) could be an efficient approach to genotype in ducks [12–14]. In this study, we carried out a genome-wide association study for feeding behavior in Pekin ducks based on GBS method to identify novel loci associated with these traits.

## Results

### Summary of phenotype and genotype

The feeding behavior information was collected from 540 ducks. The descriptive statistics of phenotypic record are shown in Table 1. The average daily feed intake (DFI) of the ducks was 0.31 kg, with a standard derivation of 0.03. Meanwhile, the average total feeding time (TFT) was 382.77 min, the mean of number of meals (NM) was 14.32, and the average meal duration (MD) was 73.41 s in ducks during the observation period.

**Table 1:** Descriptive statistics of phenotypic data

	All (*n* = 540)			
Trait	Mean	SD^**1**^	Min	Max
**DFI, kg/d**	0.31	0.03	0.22	0.39
**NM**	14.32	2.88	5.79	22.91
**MFI,g**	22.39	4.44	12.28	36.29
**MD,s**	73.41	17.28	33.59	127.25
**TFT, min**	382.77	70.7	204.85	599.48

^1^SD, standard deviation

Abbreviations: DFI, daily feed intake; NM, number of meals per day; MFI, meal feed intake; MD, meal duration per time; TFT, total feeding time

A total of 1 TB clean reads were produced and 99.15% of these were aligned to the reference genome. Through imputation and quality control, 1,899,988 SNPs were attained for association analysis. The frequency of SNPs along the genome and estimated genomic inflation factors are illustrated in supplementary data (Fig. S1, Fig. S2). The estimated genomic inflation factor fluctuated between 1.00 and 1.03, revealing good uniformity between the observed and expected distributions of the *P*-value.

### Estimation of genetic parameters

Estimation of genetic parameters using genomic information is summarized in Table 2. The heritabilities of feeding behavior traits were moderate (ranging from 0.17 to 0.32), with MD having the highest genomic heritability (0.32) among all the traits. The results showed that NM had strong negative genetic correlations with meal feed intake (MFI) and MD (ranging from − 0.91 to − 0.65), which was in accord with their phenotypic correlations. Nevertheless, the phenotypic and genetic correlations between MFI and TFT were low (ranging from − 0.06 to 0.04).

**Table 2:** Genomic heritability and genetic correlation of feeding behavior traits^1^

	DFI	NM	MFI	MD	TFT
**DFI**	**0.17 ± 0.09**	0.02 ± 0.09	0.20 ± 0.10	0.24 ± 0.18	0.32 ± 0.20
**NM**	0.27	**0.28 ± 0.10**	−0.91 ± 0.05	− 0.65 ± 0.13	0.08 ± 0.14
**MFI**	0.2	−0.85	**0.30 ± 0.11**	0.73 ± 0.11	0.04 ± 0.06
**MD**	0.01	−0.63	0.65	**0.32 ± 0.13**	0.60 ± 0.11
**TFT**	0.17	0.14	−0.06	0.59	**0.29 ± 0.11**

^1^The upper triangle showed the genetic correlation ± standard error, the lower triangle showed the phenotypic correlation, and the diagonal showed the heritability ± standard error

Abbreviations: DFI, daily feed intake; NM, number of meals per day; MFI, meal feed intake; MD, meal duration per time; TFT, total feeding time

### Association analysis for feeding behavior traits

**Fig. 1:**
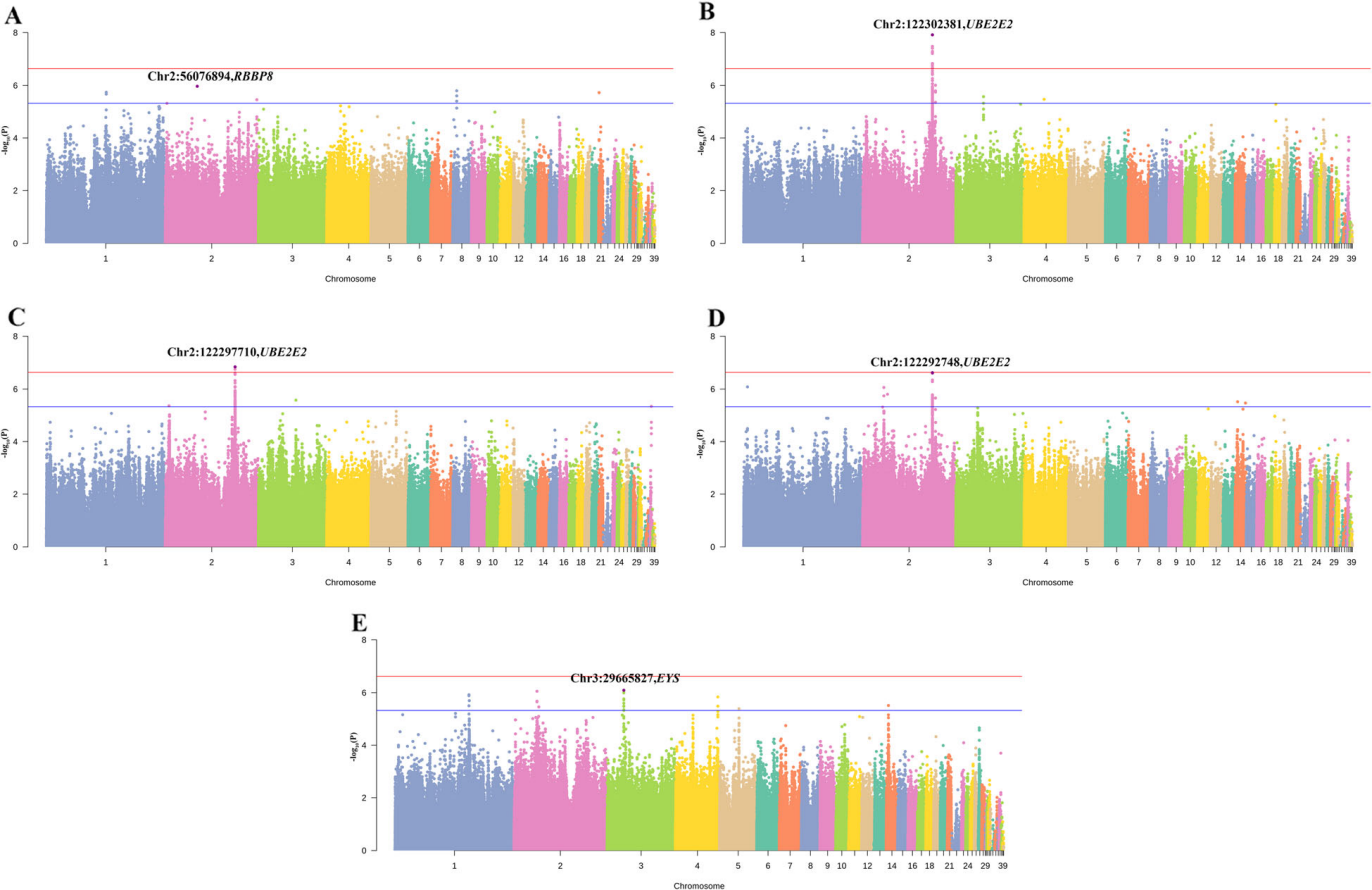
Manhattan plot for feeding behavior traits. **a** daily feed intake; **b**, number of meals per day; **c**, meal feed intake; **d**, meal duration per time; **e**, total feeding time. The horizontal red and blue lines indicate the whole-genome significance (*P*-value = 2.37E-07) and potential thresholds (*P*-value = 4.74E-06), respectively. The top SNPs were annotated with corresponding candidate genes

The results of loci-based analysis are shown in Fig. 1. A total of 30 suggestively significant loci (*P*-value < 4.74E-06) among 9 different chromosomes were identified (Table 3), including 4 loci that gained the genome-wide significance (*P*-value < 2.37E-07).

**Table 3:** information for significant SNPs associated with feeding behavior traits

Trait	N	Chr	Position	MAF	Beta	P-value	CPV(%)	Candidate gene	Distance
**DFI**	5	1	105,141,360	0.119	−3.11E-01	1.82E-06	1.46	*CLMP*	Within (intron)
		2	56,076,894	0.053	−4.75E-01	1.07E-06	1.34	*RBBP8*	13.284 kb upstream
		2	160,342,459	0.182	−2.63E-01	3.49E-06	1.01	*SNAP47*	1.798 kb upstream
		8	7,821,240	0.058	−4.58E-01	1.59E-06	1.56	*DENND1B*	Within (intron)
		21	1,442,045	0.051	4.86E-01	1.88E-06	1.67	*ARHGEF10L*	28.32 kb downstream
**NM**	8	2	122,248,468	0.302	−2.25E-01	3.27E-06	2.19	*UBE2E2*	Within (intron)
		2	122,253,614	0.373	−2.56E-01	3.34E-08	2.64	*UBE2E2*	Within (intron)
		2	122,289,379	0.291	−2.51E-01	2.31E-07	2.6	*UBE2E2*	Within (intron)
		2	122,297,710	0.354	−2.33E-01	6.52E-07	2.27	*UBE2E2*	Within (intron)
		2	122,302,381	0.302	−2.74E-01	1.21E-08	3.41	*UBE2E2*	Within (intron)
		2	127,662,769	0.227	−2.47E-01	4.41E-06	1.8	*NPVF*	78.894 kb upstream
		3	49,064,869	0.311	2.35E-01	2.66E-06	1.49	*CD164*	1.564 kb upstream
		4	35,340,778	0.106	−3.39E-01	3.37E-06	1.63	*OTUD4*	Within (intron)
**MFI**	9	2	6,590,112	0.380	−2.09E-01	4.36E-06	2.12	*KHDRBS3*	425.206 kb upstream
		2	122,248,468	0.302	2.29E-01	1.67E-06	2.7	*UBE2E2*	Within (intron)
		2	122,253,614	0.373	2.39E-01	1.81E-07	2.72	*UBE2E2*	Within (intron)
		2	122,289,379	0.291	2.42E-01	4.75E-07	2.63	*UBE2E2*	Within (intron)
		2	122,297,710	0.354	2.43E-01	1.47E-07	2.74	*UBE2E2*	Within (intron)
		2	122,302,381	0.302	2.35E-01	8.15E-07	2.73	*UBE2E2*	Within (intron)
		2	122,412,594	0.241	2.35E-01	3.05E-06	2.15	*ZNF385D*	59.762 kb upstream
		3	66,002,589	0.251	−2.39E-01	2.63E-06	2.21	*ARID1B*	226.373 kb downstream
		37	1,532,297	0.086	3.76E-01	4.56E-06	1.96	*LOC113841483*	0.38 kb upstream
**MD**	10	1	6,999,701	0.058	−4.34E-01	8.26E-07	3.14	*USP6NL*	Within (intron)
		2	37,351,206	0.270	−2.33E-01	8.7E-07	1.38	*BHLHE22*	17.535 kb downstream
		2	43,705,118	0.411	−2.11E-01	1.58E-06	1.62	*CDH2*	Within (intron)
		2	122,248,468	0.302	2.07E-01	4.7E-06	1.93	*UBE2E2*	Within (intron)
		2	122,289,379	0.291	2.14E-01	2.4E-06	1.63	*UBE2E2*	Within (intron)
		2	122,292,748	0.318	2.29E-01	2.45E-07	2.38	*UBE2E2*	Within (intron)
		2	122,302,381	0.302	2.26E-01	5.23E-07	2.46	*UBE2E2*	Within (intron)
		2	127,662,769	0.227	2.39E-01	2.2E-06	1.97	*NPVF*	78.894 kb upstream
		14	5,023,928	0.154	2.72E-01	3.04E-06	1.8	*GFPT2*	2.353 kb downstream
		14	18,817,706	0.078	3.64E-01	3.4E-06	2.21	*GABRB2*	18.24 kb downstream
**TFT**	8	1	129,679,448	0.069	−3.98E-01	1.2E-06	2.68	*EGFL6*	Within (intron)
		1	130,266,111	0.122	−3.09E-01	2.02E-06	2.35	*FRMPD4*	Within (intron)
		2	40,465,055	0.177	2.70E-01	8.85E-07	2.32	*PENK*	83.343 kb upstream
		2	43,705,118	0.410	−2.06E-01	3.48E-06	1.69	*CDH2*	Within (intron)
		3	29,665,827	0.112	3.18E-01	8.11E-07	2.65	*EYS*	221.440 kb downstream
		4	74,555,149	0.189	−2.58E-01	1.45E-06	2.07	*FAM53A*	46.599 kb downstream
		5	33,918,738	0.136	−2.77E-01	4.1E-06	2.07	*EHD4*	15.44 kb upstream
		14	4,185,488	0.108	3.15E-01	3.07E-06	2.45	*KLHL3*	180.963 kb upstream

Abbreviations: DFI, daily feed intake; NM, number of meals per day; MFI, meal feed intake; MD, meal duration per time; TFT, total feeding time. Chr, chromosome; MAF, minor allele frequency; Beta, the estimate coefficient; CPV(%), contribution of phenotypic variance explained by the SNP

For NM and MFI, a common peak of genome-wide significant SNPs was identified on chromosome 2 due to the strong genetic correlations between them (Fig. 1b, c). The top SNPs for these two traits both lay between the third and the fourth exon of ubiquitin conjugating enzyme E2 E2 (*UBE2E2)*, which could explain 3.42 and 2.74% of phenotypic variation, respectively.

As for the other feeding traits, the SNP located in the intron of *UBE2E2* on chromosome 2 was also associated with MD, accounting for 2.38% phenotypic variance. The most significant SNP for DFI was in the upstream region of RB binding protein 8 (*RBBP8)*, with a significance of 1.07E-06. Our results also revealed that the top SNP for TFT was suggested to be associated with eyes shut homolog (*EYS)*.

### Linkage disequilibrium and conditional analysis

**Fig. 2:**
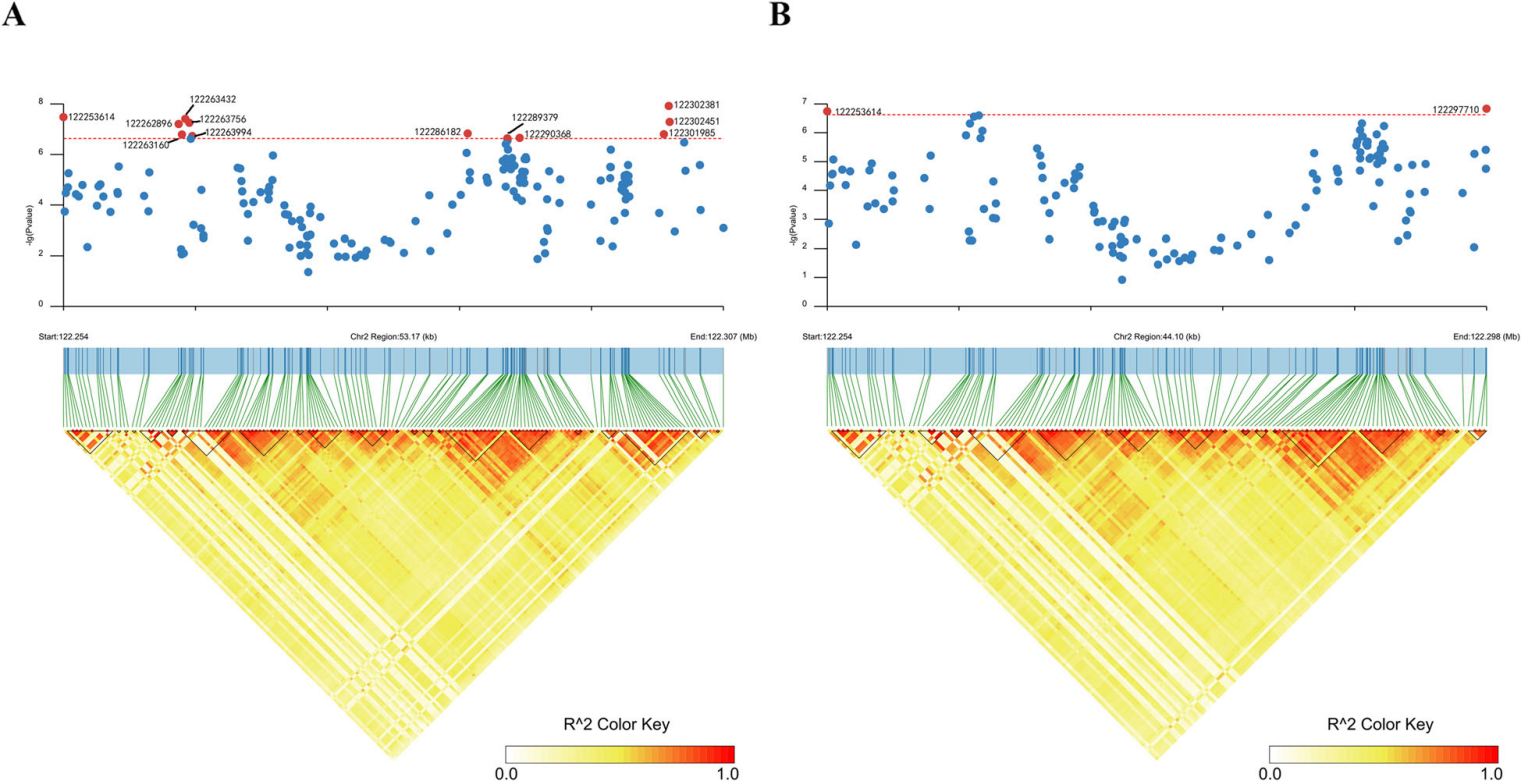
Locus-specific Manhattan plot and linkage disequilibrium analysis for genome-wide SNPs. **a**, the number of meals per day; **b**, meal feed intake. The horizontal red dash line indicates the whole-genome significance (*P*-value = 2.37E-07)

**Fig. 3:**
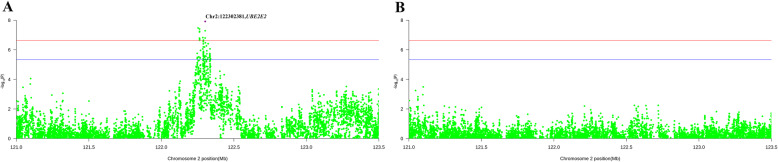
Conditional analysis on top SNP for the number of meals per day. **a**, primary association signals; **b** association results after conditional analysis. The horizontal red and blue lines indicate the whole-genome significance (*P*-value = 2.37E-07) and potential thresholds (*P*-value = 4.74E-06), respectively. The top SNPs were annotated with the corresponding candidate gene

According to Fig. 1, the tops SNPs for NM and MFI are close to each other. In order to characterize the potential relationships among them, a linkage disequilibrium analysis was performed. The results of linkage disequilibrium analysis for genome-wide significant regions are illustrated in Fig. 2. As some peak SNPs for NM were located in the same Haplotype block, we performed conditional single-marker association analysis on the top SNP to examine whether the other significant SNPs were independent of the top associated SNP, which might act as potential causal variants. Only SNPs on chromosome 2 were selected in our conditional analysis, and no additional signals were detected except for this region. The results on region-specific and conditional analysis for NM are presented in Fig. 3. The *P*-values of previous significant SNPs for NM on chromosome 2 were lower than the potential significance level. No significant association was found after conditional analysis. So we could infer that the other significant SNPs in this region gained the genome-wide significance due to their close linkage relationships with the top associated SNP.

### Functional annotation of candidate genes

A total of 25 candidate genes were detected by GWAS analysis. We performed QTL annotation of these 25 chicken ortholog genes using the chicken QTL database. Seventeen genes were annotated with QTL information in chickens. The QTL information for five genes was consistent with the feeding behavior traits collected in this study (Table 4). Rho guanine nucleotide exchange factor 10 like (*ARHGEF10L)*, which was associated with DFI in ducks in the current study, mapped to body weight QTL in chicken studies. *UBE2E2*, a common candidate gene for NM, MFI, and MD, is associated with Wattles weight QTL in chickens.

**Table 4:** Candidate gene QTL information

Gene	Associated trait	Related QTL in chicken (QTL ID)
*DENND1B*	DFI	Ileum weight QTL (96639)
*ARHGEF10L*	DFI	Body weight QTL (95430)
*GABRB2*	MD	Feather pecking QTL (137244)
*UBE2E2*	NM,MFI,MD	Wattles weight QTL (127117)
*FAM53A*	TFT	Feather pecking QTL (137261)

Abbreviations: DFI, daily feed intake; NM, number of meals per day; MFI, meal feed intake; MD, meal duration per time; TFT, total feeding time

## Discussion

### Genomic heritability

Genetic parameters play a vital role in designing the animal breeding plan, and genetic parameter estimation using the relationship matrix based on whole-genome genotypes can be an efficient way to achieve this goal [15]. As shown in Table 2, the heritability of feeding behavior traits ranges between 0.17 and 0.32. Previous work showed that the heritability of feeding behavior traits in ducks varied from 0.33 to 0.65, based on the pedigree information [11]. The findings in these two studies were consistent with one another and both of them revealed that MD had the highest heritability, arriving at 0.32 and 0.65, respectively. Howie, et al. [8] previously reported that the heritabilities of feeding behavior traits ranged from 0.30 to 0.55 in commercial broilers. Our study observed a lower estimation compared with previous studies, which might be due to the difference of genetic background and the missing heritability of rare genomic variants [16, 17].

Moreover, NM had relatively strong negative genetic and phenotypic correlations (≤ − 0.63) with MFI and MD in this study. Similar intensely negative phenotypic and genetic relationships (ranging from − 0.49 to − 0.93) were obtained in our former work [11]. It suggested that certain feeding behavior habits may affect feed consumption in ducks. Previous researches also reported that feeding behavior traits were closely associated with RFI levels while having little impact on body weight in ducks [10, 11]. Combined with these findings, selecting certain feeding behavior traits could be an effective way to achieve indirect selection for RFI without affecting body weight improvement in Pekin ducks.

### Candidate genes associated with feeding behavior

Feeding behavior traits are complex and are controlled by a collaboration of various biological processes, including energy metabolism, neurological development, and muscle system movement. In this study, *UBE2E2* is a common candidate gene for the top SNPs of NM, MFI, and MD. *UBE2E2* belongs to the *UBE2E* enzymes family and can lead to the ubiquitination of specific proteins. Mizukami, et al. [18] found that the downregulation of *UBE2E2* in rat liver cells could facilitate cell proliferation. Yaagoubi, et al. [19] discovered that *UBE2E2* accounted for metabolic syndrome in the Moroccan population. It is also reported that *UBE2E2* is involved in adipocyte development and type 2 diabetes in humans [20, 21]. Combined with these findings, we hypothesized that this gene might be involved in the regulation of body development and metabolism in fat tissue for Pekin ducks, which may rely highly on the feed consumption per meal. However, further investigation is needed.

As for DFI, two significant SNPs were detected on chromosome 2, and the candidate genes were *RBBP8* and synaptosome associated protein 47 (*SNAP47)*. Mumtaz, et al. [22] revealed that different *RBBP8* mutations led to microcephaly, intellectual disability, and short stature in Pakistani people. Li, et al. [23] found one genome-wide significant SNP marker upstream the candidate gene *RBBP8*, which was closely associated with body weight at the age of 120 days in purebred Wengshang Barred chicken. Münster-Wandowski, et al. [24] pointed out that *SNAP47* had a distinctive location in glutamatergic neurons in the mouse. In comparison with these results, we inferred that these two genes might regulate body weight gain and neuron activity in Pekin ducks. Meanwhile, one significant locus located in the intron of CXADR like membrane protein (*CLMP)* was also obtained in this study, contributing to 1.46% of the phenotypic variation. *CLMP* encodes a type I transmembrane protein and takes part in cell-cell adhesion. Previous researchers had shown that this gene was essential for normal intestinal development, and mutations in the gene were associated with congenital short bowel syndrome [25–27]. Our findings were consistent with the previous results, suggesting the latent correlation between *CLMP* and feed intake in ducks.

For TFT, the most significant SNP was located downstream of *EYS*. *EYS* was reported to play an important role in degeneration of retinitis photoreceptors in humans [28, 29]. Therefore, we hypothesized that *EYS* might be associated with visual perception in Pekin ducks. Additionally, EGF like domain multiple 6 (*EGFL6)*, cadherin 2 (*CDH2),* and EH domain containing 4 (*EHD4)* were also closely associated with TFT in our results. *EGFL6* is a member of the epidermal growth factor repeat superfamily, which coordinates the regulation of bone remodeling and glucose homeostasis in humans [30, 31]. *CDH2* produces a classical cadherin that can be crucial for bone growth and osteoblast differentiation in mice [32]. The mutation of *CDH2* is associated with knee osteoarthritis in children [33, 34]. Melo, et al. [35] reported that *EHD4* took part in the activation of ATPases in *Caenorhabditis elegans*. And *EHD2*, a paralog of *EHD4*, was identified to be differentially abundant at 4 and 24 h in postmortem normal breast and woody breast in commercial broilers (*P* < 0.05), which might imply that *EHD2* took part in the breast muscle myopathies process [36]. Integrated with these findings, we inferred that these three genes might contribute to bone development, energy metabolism and breast muscle growth in ducks, exerting an indirect impact on daily movement and feeding habits.

Moreover, as we had previously conducted a genome-wide association study for feed efficiency traits in a fat strain of Pekin ducks, Zhu et al. [37] found that interleukin 1 receptor accessory protein like 1 (*IL1RAPL1*) was associated with feed conversion ratio (FCR) and the candidate gene for residual feed intake (RFI) was ring finger protein 17 (*RNF17*). Deng et al. [13] also identified a total of 36 candidate genes for 18 carcass traits using the same flock, which revealed that ATPase phospholipid transporting 11A (*ATP11A*) was closely associated with body weight, dressed weight, eviscerated weight, foot weight, and wing weight in Pekin ducks. Compared with their results, the 25 candidate genes for feeding behavior traits in this study didn’t have any overlap with them. This might due to the fact that the genetic correlations between feeding behavior traits (including NM, MFI, MD, and TFT) and feed efficiency traits (FCR and RFI) were relatively low, which ranged from − 0.03 to 0.20 [11]. Furthermore, we selected ducks from a lean strain in this study. There were serval differences in the population’s genetic background and target traits. However, further research is needed to dissect the relationships among the candidate genes from different strains of Pekin ducks.

## Conclusions

In this study, the genetic parameters of feeding behavior traits were estimated, and the related genomic variations were identified. We obtained 30 significant SNPs using mixed-linear models, which localized to 25 candidate genes for the five different traits studied. The results of this study contributed to the sparse knowledge of feeding behavior traits in Pekin ducks and helped to achieve balanced breeding in the future.

## Methods

### Experimental population

All of the experimental ducks were reared under consistent conditions at Beijing Golden Star Duck Inc. Briefly, ducks were provided ad libitum water and commercial diets: a starter diet (from 1 day to 18 day of age) containing 19% crude protein (CP), 12.81 MJ/kg dietary metabolizable energy (ME), and a grower diet containing 17.1% CP and 11.95 MJ/kg ME during our observation period. A total of 540 19-day-old ducks (274 males and 230 females) were divided into three batches (185, 180 and 175 ducks), with an interval of 7 days between each batch. At six weeks of age, ducks were blood sampled via the caudal tibia vein after fasting for six hours.

### Collection of feeding behavior record

Combined with radio frequency identification (RFID) technology and electronic feeders, feeding information of each duck was collected during the test period. The observation period for feeding behavior started at the age of 19 days and ended at the age of 42 days. The details of the recording process and the summary of different feeding behavior traits followed the same methods in our previous research [11]. Overall, the feeding behavior traits analyzed in this study included: daily feed intake, number of meals per day, meal feed intake, meal duration per time, and total feeding time.

### Genotyping and SNP calling

Genomic DNA was extracted using a phenol-chloroform-based method, and genotyping was performed with a GBS method, which was first applied in our previous research [12]. Briefly, genomic DNA was digested with restriction endonuclease *MseI* (New England Biolab, USA). Fragments ranging from 550 to 580 bp, including adapter sequence, were sequenced using an Illumina HiSeq2500 instrument. The data were deposited in the NCBI sequence read archive (SRP155579).

Overall, a total of 1 TB clean reads were generated, together with an average sequencing depth of 1.5X for each duck. Clean data was mapped to a reference genome using BWA (v 0.7.17) [38], and the reference genome was released on Ensembl database lately (ASM874695v1 [GCA_008746955.1]). VCFtools (v 0.1.16) [39] and PLINK (v 1.90) [40] were used for quality control of the data. SNP calling was performed using the GATK HaplotypeCaller (v 4.1) [41]. All parameters were kept at default settings, except for -stand_call_conf 30. According to a high-density (sequencing depth of 10x) reference from the same population, the data were imputed using Beagle (v 5.1) [42], together with R^2^ > 0.98 for low-quality filtering. We identified a subset of tagging SNPs that passed the following thresholds: minor allele frequency (MAF) > 0.05, sample call rate ≥ 0.95, and SNP call rate ≥ 0.95 with PLINK.

### Statistical analysis

Normality test was processed using the Shapiro-Wilk test to check the distribution of phenotypes. If the traits were skewed from the normal test, the phenotypic data were normalized by the rank-transformation, following a standard normal distribution with a mean of 0 and a standard deviation of 1. The independent SNP set was used via the PLINK command (−-indep-pairwise 25 5 0.2) for principal component analysis (PCA) (Fig. S3), and the top three eigenvectors were implemented in association analysis to account for the effect of population stratification. The effects of covariates, including sex and batch, on quantitative phenotypes were assessed with analysis of variance (ANOVA) using R software (v 3.5.3; https://mirrors.tuna.tsinghua.edu.cn/CRAN/, TUNA Team, Tsinghua University, Beijing, China), and covariates with *P*-value < 0.05 were included in a mixed linear regression model as the fixed effects (Table S1).

The association analyses were performed using leaving-one-chromosome-out (LOCO) algorithm in GCTA (v 1.26.0) [43], which implements a mixed linear model with the chromosome where the candidate SNP is located excluded from the calculation of the genomic relationship matrix (GRM). The model was conducted as following:

$$ \mathrm{y}=\mathrm{a}+\mathrm{BX}+\mathrm{g}+\mathrm{e} $$

Where y is a vector of the normalized phenotype, a is the general mean, X is the matrix of fixed effects (batch, sex and eigenvectors derived from PCA), B is the vector of corresponding coefficients; g is the accumulated effect of all SNPs except those on the chromosome where the candidate SNP is located (the variance of g will be re-estimated each time when a chromosome is excluded from calculating the GRM), e is the vector of random residuals.

The SNP-based genetic parameters for feeding behavior and the contribution of the phenotypic variances for significant SNPs were estimated using the GREML algorithm in GCTA (v 1.92.2) [44]. The linear mixed model was performed as follows:

$$ \mathrm{y}=\mathrm{Wb}+\mathrm{Zu}+\mathrm{e} $$

$$ \mathrm{u}\sim N\ \left(0,\mathrm{I}{\sigma}_u^2\right),\mathrm{e}\sim N\ \left(0,\mathrm{I}{\sigma}_e^2\right) $$

var$$ \left(\mathrm{y}\right)=\mathrm{N}{\sigma}_u^2+\mathrm{I}{\sigma}_e^2={\sigma}_G^2+{\sigma}_e^2,{h}_{SNP}^2={\sigma}_G^2/\left({\sigma}_G^2+{\sigma}_e^2\right) $$

Where y is a vector of phenotypic value, W is the matrix of fixed variables, and Z represents the matrix of random variables, which refers to the effects of all SNPs on phenotype; e stands for random residuals, I is an identity matrix, b and u are the corresponding coefficients of fixed and random effects, respectively; ${\sigma}_u^2$, ${\sigma}_e^2$ and ${\sigma}_G^2$ are the variances for random effects, residual effects and total additive genetic effects, respectively; ${h}_{SNP}^2$ is heritability based on the genomic information.

The genomic inflation factor (**λ**) was calculated with R package qqman (0.1.4) [45]. Bonferroni correction was performed to establish proper thresholds for genome-wide potential and significant associations. The independent locus number was calculated by the simpleM method [46]. Therefore, the 5% genome-wide significance level was 2.37E-07 (0.05/211170) and the potential significance level was 4.74E-06 (1/211170).

### Conditional analysis

The conditional analysis was conducted to examine the potential associated SNPs that might be masked by a strong signal. Briefly, the initial region was tested with the strongest SNP covariate. Then, association analysis conditioning on the selected SNP was implemented to search for the top SNP iteratively one by one via a stepwise model selection procedure until no SNP had a conditional *P*-value that passed the significance level. GWAS does not distinguish a genuine causal locus from those statistically significant loci within a strong linkage disequilibrium (LD) region. Therefore, in order to characterize potential candidate genes responsible for a trait, we conducted a LD analysis and inferred the haplotype blocks containing peak SNPs by LDBlockShow [47].

### Functional annotation

For all the SNPs that exceeded a potential significant threshold (*P*-value < 4.74E-06), we looked for the nearest candidate genes using BEDTools [48]. Due to the lack of a duck QTL database, the QTL information of candidate genes was extracted from the AnimalQTLdb [49] using chicken orthologs.

## Supplementary Information

**Additional file 1 Table S1.**

**Additional file 2 Fig. S1**-**Fig. S3.**

## Data Availability

All data generated or analyzed during this study are included in this article and the additional files. The raw duck GBS data was deposited in Sequence Read Archive (SRA) database under the accession numbers SRP155579 (https://www.ncbi.nlm.nih.gov/sra/?term=SRP155579).

## Abbreviations

DFI: daily feed intake
NM: number of meals per day
MFI: meal feed intake
MD: meal duration per time
TFT: total feeding time
GBS: genotyping-by-sequencing
GO: gene ontology
GWAS: genome-wide association study
LD: linkage disequilibrium
SNP: single nucleotide polymorphism
