# Supplementary material for: Genome-wide association study reveals novel loci associated with feeding behavior in Pekin ducks
Source: BMC Genomics. 2021 May 8;22:334. doi: 10.1186/s12864-021-07668-1 (PMC8106866; doi:10.1186/s12864-021-07668-1)

**Title**

**Genome-wide association study reveals novel loci associated with feeding behavior in Pekin ducks**

Guang-Sheng Li^*^, Feng Zhu^*^, Fan Zhang^*^, Fang-Xi Yang^†^, Jin-Ping Hao^†^,Zhuo-Cheng Hou^*,1^

** National Engineering Laboratory for Animal Breeding and Key Laboratory of Animal Genetics, Breeding and Reproduction, MARA; College of Animal Science and Technology, China Agricultural University, Beijing, China 100193*

*†Beijing Golden Star Inc., Beijing, China 100076*

**^1^Corresponding author: Zhuo-Cheng Hou**

**E-mail address:** [**zchou@cau.edu.cn**](mailto:zchou@cau.edu.cn)

**Additional file 2:**

**Figure S1. Circus plot showing distribution of SNPs on the duck genome.** The inner histogram plot presents the frequency of SNPs in 1Mb windows along the genome. The outer tracks display the candidate genes associated with significant SNPs.

**Figure S2. Quantile-quantile (QQ) plot for feeding behavior traits.** (A) daily feed intake; (B), number of meals per day; (C), meal feed intake; (D), meal duration per time; (E), total feeding time.

**Figure S3. Principal component analysis of Pekin ducks.**


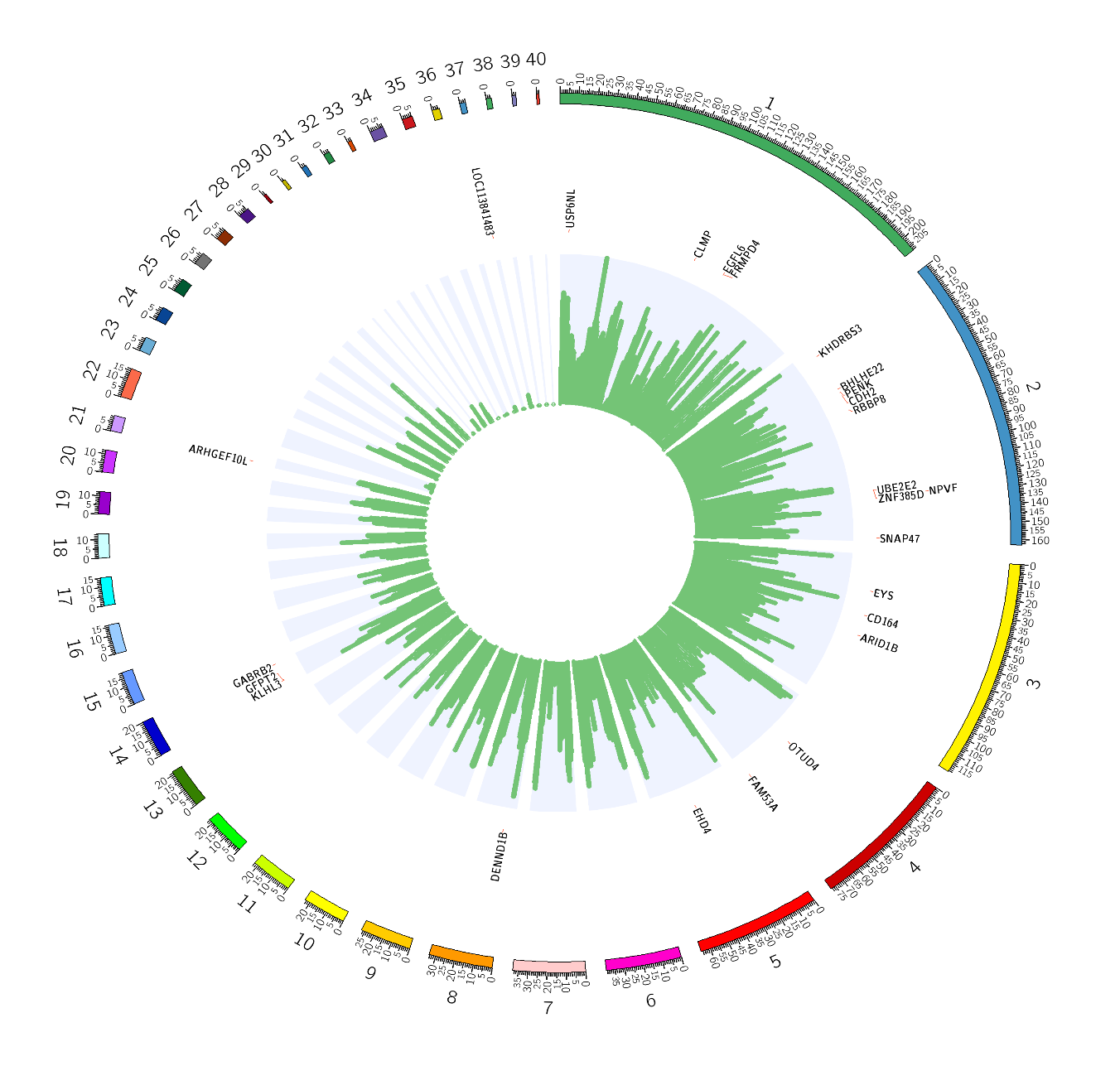
**Figure S1**


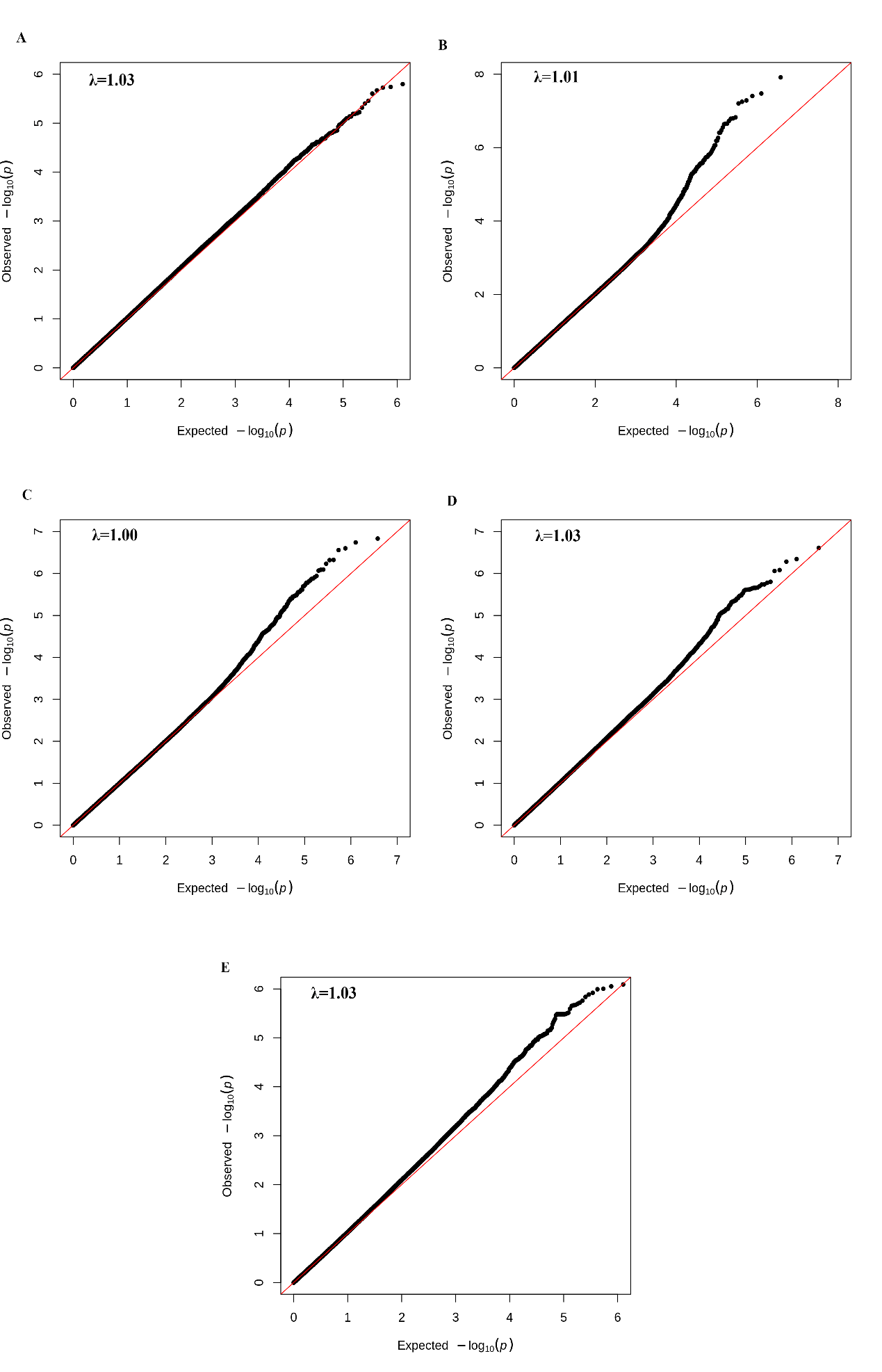
**Figure S2**

**Figure S3**


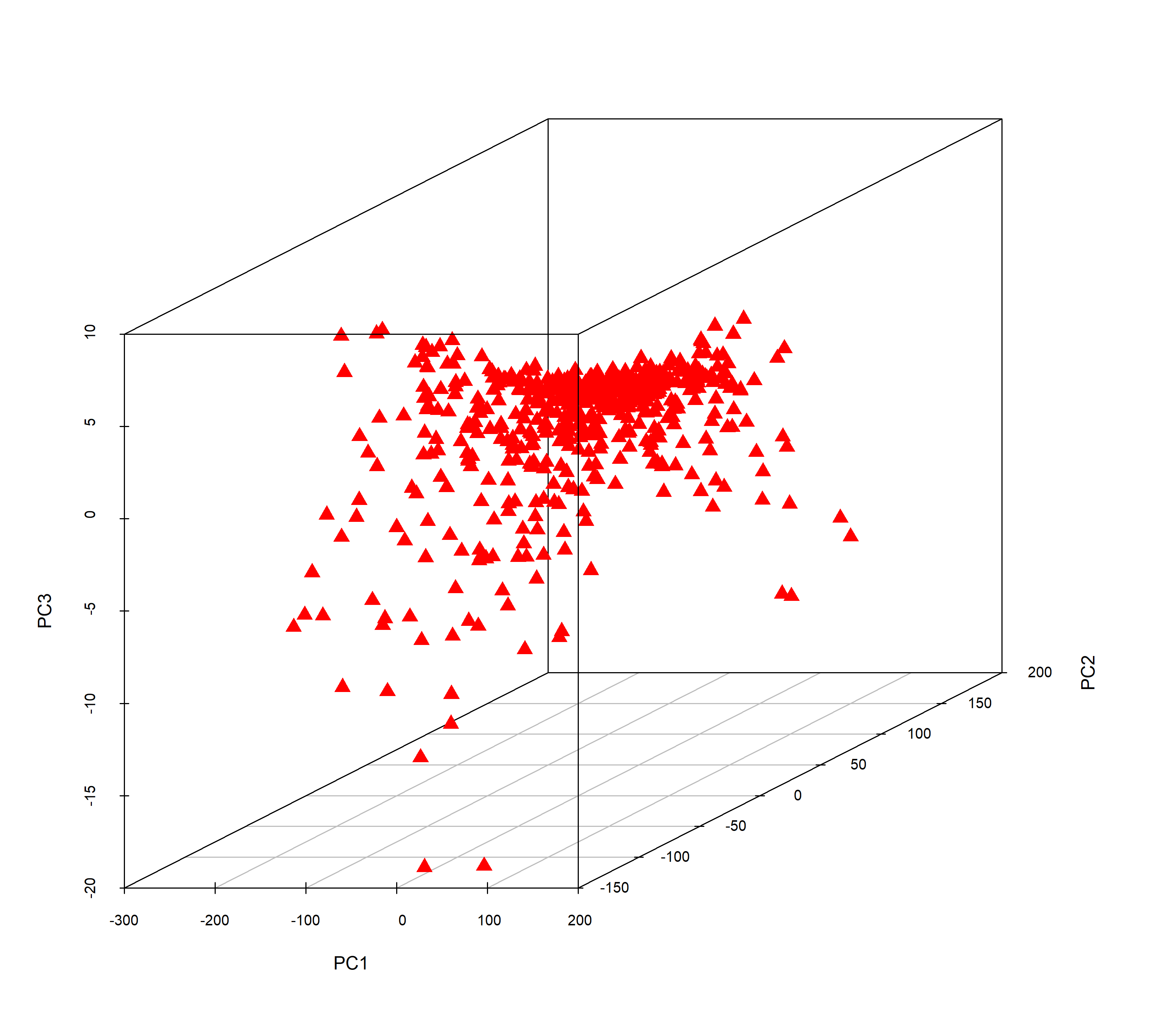

Supplement: Supplementary file 2 — Additional file 2 Fig. S1-Fig. S3. [file 12864_2021_7668_MOESM2_ESM.docx]
